# Neural control of redox response and microbiota-triggered inflammation in *Drosophila* gut

**DOI:** 10.3389/fimmu.2023.1268611

**Published:** 2023-10-26

**Authors:** Naoyuki Fuse, Haruka Hashiba, Kentaro Ishibashi, Takuro Suzuki, Quang-Dat Nguyen, Kiho Fujii, Wakako Ikeda-Ohtsubo, Haruki Kitazawa, Hiromu Tanimoto, Shoichiro Kurata

**Affiliations:** ^1^ Graduate School of Pharmaceutical Sciences, Tohoku University, Sendai, Japan; ^2^ Graduate School of Agricultural Science, Tohoku University, Sendai, Japan; ^3^ The Division for the Establishment of Frontier Sciences of the Organization for Advanced Studies, Tohoku University, Sendai, Japan; ^4^ Graduate School of Life Sciences, Tohoku University, Sendai, Japan

**Keywords:** *Drosophila*, gut, neuron, immunity, RNA-Seq

## Abstract

**Background:**

The neural system plays a critical role in controlling gut immunity, and the gut microbiota contributes to this process. However, the roles and mechanisms of gut-brain-microbiota interactions remain unclear. To address this issue, we employed *Drosophila* as a model organism. We have previously shown that NP3253 neurons, which are connected to the brain and gut, are essential for resistance to oral bacterial infections. Here, we aimed to investigate the role of NP3253 neurons in the regulation of gut immunity.

**Methods:**

We performed RNA-seq analysis of the adult *Drosophila* gut after genetically inactivating the NP3253 neurons. Flies were reared under oral bacterial infection and normal feeding conditions. In addition, we prepared samples under germ-free conditions to evaluate the role of the microbiota in gut gene expression. We knocked down the genes regulated by NP3253 neurons and examined their susceptibility to oral bacterial infections.

**Results:**

We found that immune-related gene expression was upregulated in NP3253 neuron-inactivated flies compared to the control. However, this upregulation was abolished in axenic flies, suggesting that the immune response was abnormally activated by the microbiota in NP3253 neuron-inactivated flies. In addition, redox-related gene expression was downregulated in NP3253 neuron-inactivated flies, and this downregulation was also observed in axenic flies. Certain redox-related genes were required for resistance to oral bacterial infections, suggesting that NP3253 neurons regulate the redox responses for gut immunity in a microbiota-independent manner.

**Conclusion:**

These results show that NP3253 neurons regulate the appropriate gene expression patterns in the gut and contribute to maintain homeostasis during oral infections.

## Introduction

The gut is a central immune tissue that is frequently exposed to various pathogens ingested with food. The gut absorbs nutrients from food but excludes the pathogens. In addition, the gut harbors commensal bacteria known as microbiota, which benefit the host (e.g., bacteria produce metabolites that can be utilized as nutrients). Under these circumstances, the gut performs the seemingly contradictory tasks of preventing the growth of pathogens via immune response and allowing the growth of commensal bacteria for immune tolerance (1, 2). To maintain this balance, gut immunity is precisely regulated by complex mechanisms. Intestinal epithelial cells express mucin in the lumen, forming a physiological barrier against pathogens and enhancing their clearance. Furthermore, the gut produces antimicrobial peptides (AMPs) and reactive oxygen species (ROS) that directly kill pathogens. Cytokines secreted by intestinal and surrounding immune cells modulate the systemic inflammatory status. In addition, the microbiota contributes to gut immunity by secreting metabolites that inhibit pathogen growth and by competing with pathogens for essential nutrients (3). These molecular pathways work together as a gut immunity system.

It is widely known that the nervous system is involved in regulating gut immunity. Numerous neurons in the gut form a network called the enteric nervous system (ENS) (4, 5). Although the ENS can function independent of the brain, it essentially cooperates with the brain to sense and control the physiological status of the gut. The mammalian vagus nerve consists of afferent and efferent neurons between the ENS and the brain. A previous study showed that pathogen-derived metabolites induce gut epithelial cells to secrete serotonin, activating the ENS and the vagus nerve (6). In addition, neuron-derived acetylcholine acts on intestinal macrophages to induce the suppression of inflammatory responses (7). Furthermore, some studies have shown that the nervous system is involved in controlling gut microbiota (8, 9). For example, neuron-derived norepinephrine directly stimulates the quorum-sensing pathway of the microbiota via inter-kingdom signaling (10). Thus, the brain-gut-microbiota interaction is essential for maintaining gut homeostasis, and its dysfunction causes gut diseases, such as irritable bowel syndrome. Owing to its importance in health and disease, brain-gut-microbiota interaction has been extensively studied over the last decade; however, its underlying mechanism is unclear.

To investigate this issue, *Drosophila* is a suitable model organism (11–13). The structure and function of the gut are conserved between *Drosophila* and mammals. *Drosophila* gut consists of several cell types (e.g., epithelial cells, endocrine cells, and intestinal stem cells); it is divided into functional compartments (e.g., clot, midgut, and hindgut), similar to that of mammals. Compared with the thousands of species in the human microbiota, *Drosophila* microbiota is relatively simple, with only a few species (e.g., *Lactobacillus* sp. and *Acetobacter* sp.). *Drosophila* neural system consists of 100,000 neurons and is much simpler than that of humans. Various genetic tools are available for studying neuronal function in *Drosophila*. The FlyLight project has generated 7,000 transgenic lines in which Gal4 is expressed in a subset of neurons, and the collection of Gal4 drivers covers almost all fly neurons (14). As Gal4 drivers induce the expression of any gene under UAS control, the morphology of the neurons can be observed based on the expression of fluorescent proteins (e.g., GFP and RFP), and neuronal activity can be modified based on the expression of neural inhibitors (e.g., Kir2.1 and Shi^ts^) or activators (e.g., dTrpA1 and Chrimson). The physiological functions of a given neuron can be characterized using Gal4 driver lines. For example, Ilp7-positive neurons sense the amount of food in the gut and control the feeding behavior of flies (15). Akh (*Drosophila* homolog of glucagon)-positive neurons sense nutritional conditions in the gut and control lipid metabolism in fat body cells (16).

We have previously screened Gal4 enhancer trap lines (17) to search for neurons involved in gut immunity and identified the NP3253 line (18). When the activity of Gal4-expressing neurons in the NP3253 line (termed NP3253 neurons hereafter) was genetically inhibited by Kir2.1 (a mammalian inwardly rectifying K^+^ channel) expression, these flies (NP3253-Gal4, UAS-Kir2.1 flies referred to as NP3253>Kir2.1 flies) became susceptible to oral infection by the gram-negative bacterium *Erwinia carotovora carotovora 15* (*Ecc15*). However, feeding and excretory behaviors were normal in NP3253>Kir2.1 flies, suggesting their roles in gut immunity rather than feeding behaviors. Although NP3253 neurons consist of dozens of neurons located in the brain and anterior midgut (proventriculus), Gal4-expressing cells in the NP3253 lines were also observed in non-neural cells (e.g., tracheal cells and some intestinal epithelial cells). Therefore, the phenotypes of the NP3253>Kir2.1 fly might be attributed to Kir2.1 expression in non-neural cells. To exclude this possibility, we used elav-Gal80 to inhibit Gal4 activity in pan-neural cells (19) and observed that elav-Gal80 rescued the susceptibility to oral infection in NP3253>Kir2.1 flies. This suggests that NP3253 neurons are essential for resistance to oral infections. However, it is unclear how NP3253 neurons control gut immunity.

In this study, we aimed to evaluate the role of NP3253 neurons in the regulation of gut immunity. We performed RNA-Seq analysis of the gut and compared the gene expression between NP3253>Kir2.1 and control flies. In addition, we examined the involvement of the microbiota in gut gene expression using axenic flies. We characterized the gut gene expression regulated by NP3253 neurons and microbiota.

## Materials and methods

### Fly stocks

*Drosophila melanogaster* lines used in this study are as follows. The NP3253-Gal4 and NP1-Gal4 lines were obtained from Kyoto Drosophila Stock Center. The UAS-Kir2.1::EGFP (20), tubP-Gal80ts, and UAS-mCD8::GFP lines were obtained from the Bloomington Drosophila Stock Center (BDSC). UAS-dTrpA1 was gifted by P. Garrity (21). elav-Gal80 was gifted by Y. Jan (19). Flies were reared at 18 or 25°C in plastic vials containing standard cornmeal-agar medium. To inhibit or activate neural activity, adult flies of NP3253-Gal4/tubP-Gal80ts; UAS-Kir2.1::EGFP/+ (NP3253>Kir2.1 flies) or NP3253-Gal4/UAS-dTrpA1 (NP3253>dTrpA1 flies) were reared at 29 or 30°C for 2 days before experiments. Adult flies of NP3253-Gal4/tubP-Gal80ts; UAS-mCD8::GFP/+ (NP3253>GFP flies) were used as controls. The following RNAi lines were obtained from the Vienna Drosophila Resource Center (VDRC): UAS-Cyp6d5 RNAi (#107641), UAS-Sodh-1 RNAi (#3761), and UAS-Cyp313b1 RNAi (#102986). UAS-GFP RNAi was obtained from BDSC (#9331) and used as the control. Adult flies of NP1-Gal4, UAS-XX RNAi (NP1 > XX RNAi flies) were used for the knockdown experiment. Female flies were used in all of the following experiments.

### Oral bacterial infection

*Pseudomonas entomophila* (*Pe*) was a gift by S. Kawabata (22). The *Pe* bacteria were grown in LB broth (Nacalai Tesque) at 29 or 30°C overnight. The bacterial culture was centrifuged at 5,000 rpm for 10 min. The bacterial pellet was resuspended in 2.5 or 5% sucrose indicated in the experiment (bacteria solution). The cell concentration was adjusted to an OD600 of 20, 10 (for the survival assay), 5 (for the bacterial load assay), or 0.5 (for RNA-seq). For oral infection, flies were starved for 2 h at 29 or 30°C and placed in a culture vial with a filter paper containing bacterial solution for a day.

### Bromophenol blue feeding assay

The assays were performed as previously described (23). To quantify feeding, five female flies were starved for 2 h at 30°C and fed with 5% sucrose solution containing 0.5% bromophenol blue (BPB) sodium (Sigma) for 30 min. The flies were placed in new vials containing normal cornmeal medium for 1 h to quantify the excretion rate. Subsequently, they were placed in a tube and homogenized in 50 µL Milli-Q water with a pestle. Fly extracts were centrifuged twice (14,600 x g, 2 min, 4 °C) to remove debris. The absorbance at 594 nm was measured using a NanoDrop spectrophotometer (Thermo Fisher Scientific).

### Bacterial load assay

To measure the *Pe* bacterial load, the flies were collected 24 h after *Pe* oral infection and were washed briefly with 70% ethanol. Subsequently, one fly was placed into a tube and homogenized using a pestle in 100 μL of LB broth. The fly extract was serially diluted, and 10 μL of the diluted extract was spotted onto LB agar plates. The plates were incubated at 30 °C overnight. The bacterial load in the original fly extract was calculated and expressed as colony-forming units per fly (CFU/fly). In the context of negative control, we observed a lack of colony formation in the flies without *Pe* infection. This confirmed the absence of colony formation by commensal bacteria under this condition.

To measure the commensal bacterial load, the conventional or axenic-reared flies were collected (see Axenic rearing section) and washed briefly with 70% ethanol. Five flies were placed in a tube and homogenized using a pestle in 500 μL of LB medium. One hundred microliters of the extract were spread on MRS (BD Difco) agar plates. The plates were incubated at 30 °C for 3 days, and colony numbers were counted. We usually observed numerous colonies in conventionally reared flies, but absence of colony in axenic reared flies.

### Sample preparation for RNA-seq

For EXP1 (see Results section), NP3253>GFP (control), NP3253>dTrpA1 (neuron-activated), NP3253>Kir2.1 (neuron-inactivated), and elav-Gal80, NP3253>Kir2.1 (neuron-rescued) flies were collected. To inactivate or activate NP3253 neurons, adult female flies were reared on cornmeal medium at 29 °C for 2 days and were starved for 3 h. Then, flies were fed *Pe* or sucrose solution for 1 day, and the guts were dissected. As NP3253>Kir2.1 flies are susceptible to *Pe* infection, we used a low concentration of *Pe* (OD = 0.5) for RNA-seq experiment.

For EXP2 (see Results section), NP3253>GFP (control) and NP3253>Kir2.1 (neuron-inactivated) flies were collected under conventional (CV) and germ free (GF) conditions (see Axenic rearing section). To inactivate NP3253 neurons, the conventional and axenic flies were reared on normal and sterile cornmeal medium, respectively, at 30 °C for 2 days, and the guts were dissected.

For both EXP1 and EXP2, three biological replicates were prepared for each experimental condition. Total RNAs were prepared from 10-15 guts of each sample using the TRIzol reagent (Thermo Fisher Scientific). The yield and purity of the RNAs were evaluated using the NanoDrop (Thermo Fisher Scientific), Qubit (Invitrogen), and Bioanalyzer (Agilent). Sequencing libraries were prepared using a strand-specific RNA library prep kit (Agilent Technologies). The Illumina HiSeq 2000 system was used for sequencing (100 bases, single-end). Raw data of sequences were deposited on DDBJ, DRA (accession number DRA008209 and DRA012434).

### RNA-seq data analysis

Transcriptome analyses were performed using Linux or Macintosh operating systems as described previously (24). Analyses were performed using the NIG supercomputer at the National Institute of Genetics (ROIS). Adaptor sequences (Illumina TruSeq Adaptors) were removed from the read sequences (fastq files) using Cutadapt. The cleaned read sequences were mapped to the *D. melanogaster* reference genome (ver. 6.04) using the Hisat2 software. The number of reads was counted for each gene from the gene annotation data (ver. BDGP6.79) using HtSeq analysis. Genes showing an extremely low or high expression level (average read count was < 10 or > 100,000 for EXP1; < 3 or > 100,000 for EXP2) were eliminated from the subsequent analyses.

Read count data were analyzed using the R environment (version 3.6.0). A multidimensional scaling (MDS) analysis was performed using the edgeR package. Differentially expressed genes (DEGs) were identified using a statistical criterion (FDR-adjusted p-value was < 0.05, and fold change was > 2 or < 0.5). Z-scores of the DEGs were calculated using the Scrime package. Cluster analysis of DEGs was performed using the gplots package. The web-based databases DAVID (25) and FlyBase (https://flybase.org/) were used to analyze Gene Ontology (GO).

### RT-qPCR

The expression levels of *Diptericin A* (*DptA*) and *rp49* were determined using RT-qPCR as described previously (24). Adult females were reared at 29 °C for 2 days, and the guts were dissected. Total RNA was extracted from 10 guts for each sample, and three samples were analyzed as biological replicates for each experimental condition. All samples were derived from a single batch experiment. Complementary DNA (cDNA) was synthesized from total RNA using ReverTra Ace (Toyobo), according to the manufacturer’s instructions. RT-qPCR was performed using FastStart DNA Master SYBR Green I (Roche) on the LightCycler ST300 (Roche). The primers used for the analysis were *DptA* Fw (5′- GTTCACCATTGCCGTCGCCTTAC-3′), *DptA* Rv (5′CCCAAGTGCTGTCCATATCCTCC-3′), *rp49* Fw (5′- AGATCGTGAAGAAGCGCACCAAG-3′), and *rp49* Rv (5′- CACCAGGAACTTCTTGAATCCGG-3′). Copy numbers were calculated using the data obtained from standard plasmids carrying PCR products.

### Axenic rearing

Axenic flies were prepared as previously described (26). Briefly, embryos were collected and washed with Milli-Q water and 70% EtOH. Subsequently, they were dechorionated with 2.7% sodium hypochlorite for 1 min and washed with Milli-Q water, 70% EtOH, and sterile Milli-Q water. Then, embryos were transferred to a sterilized cornmeal medium and reared until eclosion. Axenic preparation according to this method was confirmed by the absence of bacteria in the bacterial load assay, as described above.

For the survival assay of the axenic flies, adult females reared at 18°C were transferred to a sterile cornmeal medium at 30°C; surviving flies were counted for 5 days. Concurrently, conventional flies were reared on a nonsterile cornmeal medium. For RNA-seq analysis, the guts were dissected from these flies as described above.

### H_2_O_2_ feeding

For the survival assay, hydrogen peroxide (Kanto Chemical) was diluted to 0.6% with a 5% sucrose solution. To inhibit neural activity, 5 to 8-day-old flies were reared at 29°C for 2 days and were starved for 2 h. Subsequently, the flies were placed in a culture vial with a filter paper containing 500 μL of 0.6% H_2_O_2_ or 5% sucrose solution for 3 days (exchanging fresh vials daily). After feeding, flies were transferred to a normal or sterile cornmeal medium, and the surviving flies were counted for the following 3 days.

### Statistical analysis

R program (http://www.r-project.org/) (version 3.6.0) was used for all statistical analyses. The bacterial loads were statistically compared using the Kruskal–Wallis analysis of variance (ANOVA), followed by the Wilcoxon rank-sum test. Gene expression analyses for RNA-seq data and RT-qPCR data were performed using ANOVA, followed by Tukey’s HSD *post-hoc* test.

## Results

### NP3253 neurons regulate the susceptibility to pathogens

**Figure 1:**
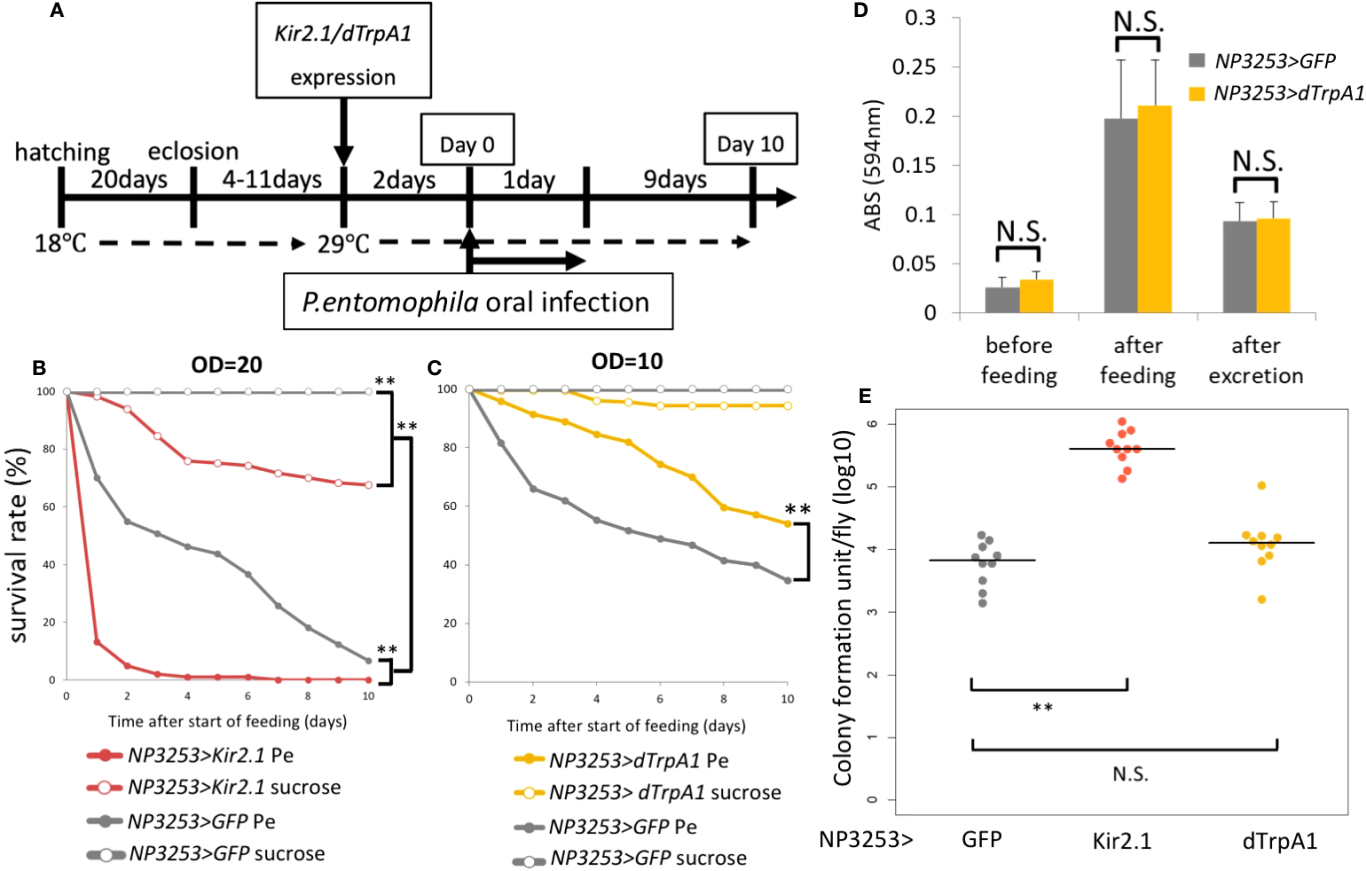
Roles of NP3253 neurons in oral bacterial infection **(A)** Timetable for oral infection experiment. The expression of Kir2.1 was induced by inactivating temperature-sensitive Gal80 (Gal80ts) at 29°C for 2 days. Neural activation using dTrpA1 was induced by the same condition. After then (day 0), the flies were fed with *Pe* for 1 day and were moved to the standard medium. Survival rate was monitored for 10 days. **(B)** Survival curves of NP3253>GFP or NP3253>Kir2.1 flies orally infected with *Pe* (OD=20). **(C)** Survival curves of NP3253>GFP or NP3253>dTrpA1 flies orally infected with *Pe* (OD=10). Numbers of flies used in these experiments are **(B)** 93, 118, 120, and 181 flies and **(C)** 284, 208, 198, 180 flies/6-9 vials (*Pe*- and sucrose-fed NP3253>Kir2.1 **(B)** or dTrpA1 **(C)** flies, and *Pe*- and sucrose-fed NP3253>GFP flies, respectively). Asterisks indicate statistical significance (p-value < 0.01) in the Log-rank test. **(D)** Feeding assay with BPB-containing food. The food intake and excretion rates were evaluated based on ABS (594 nm) of the fly extract after feeding and excretion, respectively. The differences between NP3253>dTrpA1 flies and NP3253>GFP flies were not significant (N.S.) in the Student’s t-test. **(E)** Bacterial load assay for orally *Pe*-infected flies. The flies were collected 24 h after infection. Each dot represents data from an individual. Asterisks and N.S. indicate statistical significance (p-value < 0.01) and non-significance, respectively, in Kruskal-Wallis ANOVA and Wilcoxon rank sum test.

We previously showed that NP3253> Kir2.1 flies are susceptible to the bacterium *Ecc15* by oral infection. We investigated whether this susceptibility could be extended to other bacteria. Although *Ecc15* is not pathogenic to the wild-type *Drosophila* line, we examined susceptibility to the highly pathogenic bacterium, *Pe* (27). In our experimental system using Gal80ts (see Materials and Methods), NP3253 neurons were inactivated by Kir2.1 expression after a temperature shift (**Figure 1A**). Two days after neural inactivation, flies were fed the bacterial solution (or sucrose solution) for 1 day, and dead flies were counted daily for 10 days. As expected, *Pe* was highly toxic even in NP3253 >GFP flies (hereafter referred to as control flies). In addition, the survival rate under *Pe* infection was significantly lower in NP3253>Kir2.1 flies than in control flies (**Figure 1B**). These results suggest that NP3253>Kir2.1 flies are susceptible to oral bacterial infections, probably regardless of the bacterial species.

Next, we examined the phenotype of NP3253>dTrpA1 flies, in which NP3253 neurons were hyperactivated. Under *Pe* infection, the survival rate of NP3253>dTrpA1 flies was significantly higher than that of the control flies (**Figure 1C**). This suggests that manipulating NP3253 neurons may positively or negatively affect susceptibility to oral infection. To examine feeding behavior, we measured food intake and excretion of BPB-containing foods. We detected an increase and decrease in the amount of BPB in fly bodies after feeding and excretion, respectively. There was no difference in the amount of BPB between NP3253>dTrpA1 and control flies (**Figure 1D**), indicating that hyperactivation of NP3253 neurons did not alter feeding behavior. We also measured the bacterial load in fly bodies 24 h after oral infection with *Pe*. The *Pe* load was higher in NP3253>Kir2.1 flies than in control flies, suggesting that the activity of NP3253 neurons is required to suppress the growth of *Pe* in flies. In contrast, NP3253>dTrpA1 flies showed a similar *Pe* load as that of control flies, suggesting that hyperactivation of NP3253 neurons does not further suppress the growth of *Pe*. We hypothesized that NP3253 neurons contribute to tolerance (protecting the host from pathogen-induced damage) and resistance (suppressing bacterial growth) to oral infection.

### Transcriptional profiling associated with the activity of NP3253 neurons and oral bacterial infection

To investigate how NP3253 neurons control gut physiology during oral infection, we performed RNA-seq analysis of the gut. Adult female flies were fed *Pe* or sucrose solution for 1 day. The guts were dissected from the following seven fly conditions (feeding conditions + genotypes): *Pe*- or sucrose-fed NP3253>GFP (control) flies, *Pe*- or sucrose-fed NP3253>dTrpA1 (neuron-activated) flies, *Pe*- or sucrose-fed NP3253>Kir2.1 (neuron-inactivated) flies, and sucrose-fed elav-Gal80, NP3253>Kir2.1 (neuron-rescued) flies. Total RNAs were extracted from guts and subjected to RNA-Seq analysis (**Table S1**, **Data S1**). This analysis was termed EXP1 because we performed an additional RNA-seq analysis, as described later.

**Figure 2:**
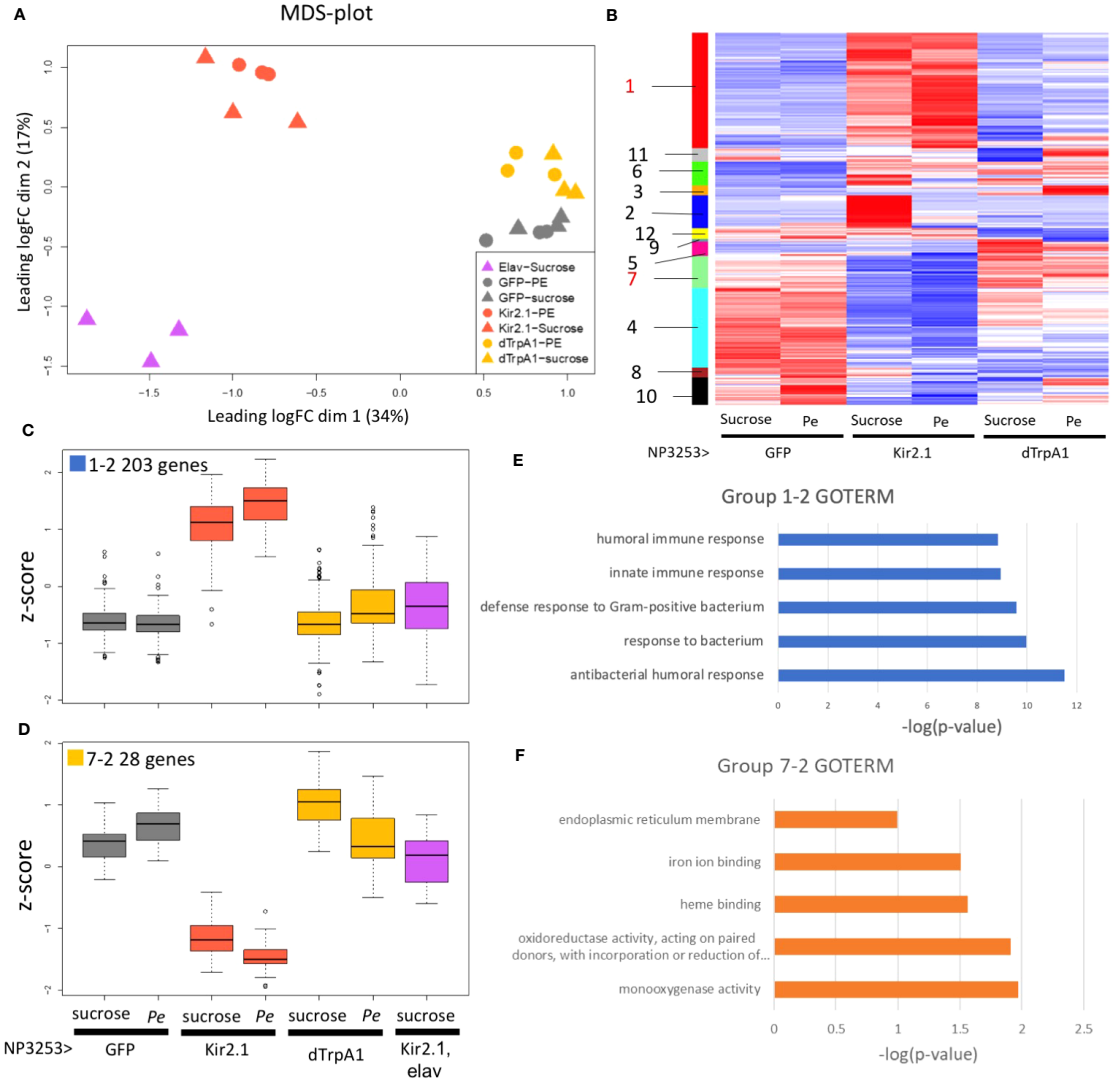
RNA-seq analysis under *Pe*-infection conditions (EXP1) **(A)** MDS plots of transcriptomes for all samples (EXP1). Proximity between plots correlates to the similarity of transcriptomes between samples. **(B)** Heat maps of z-scores for 1139 DEGs. Each column represents data from NP3253>GFP, NP3253>Kir2.1, and NP3253>dTrpA1 flies with or without *Pe* infection. Genes (rows) were arranged based on cluster analysis, and cluster groups are indicated as color bars and numbers on the left. **(C, D)** Box plots of z-scores of DEGs categorized into groups 1-2 **(C)** and 7-2 **(D)**, including data from elav-Gal80, NP3253>Kir2.1 flies. **(E, F)** GO analysis for groups 1-2 **(E)** and 72 **(F)**. GO terms are listed in Biological Process **(E)** and Molecular Function **(F)**. Bar graphs show the p-values of each GO term.

MDS analysis was performed to examine the similarity of overall gene expression between samples (**Figure 2A**). Replicate samples under the same fly conditions were plotted closely, indicating the validity of our RNA-Seq. The *Pe*- and sucrose-fed samples were plotted closely for each fly genotype, suggesting that the effect of *Pe* infection was relatively low. When comparing fly genotypes, NP3253>Kir2.1 flies showed more significant changes from control flies than NP3253>dTrpA1 flies. This finding suggests that inhibition of NP3253 neurons may have a more significant effect on the gut than neuronal activation.

We hypothesized that elav-Gal80, NP3253>Kir2.1 flies would show similar gene expression to the control flies because neuronal inactivation is rescued by elav-Gal80. However, flies of this genotype showed more altered gene expression than those of the other genotypes (**Figure 2A**). We then consider the possibility that the elav-Gal80 line might have a genetic background that strongly affects gene expression. Therefore, we did not use the data from elav-Gal80, NP3253>Kir2.1 flies for overall gene expression analysis (e.g., **Figure 2B**) but used them to analyze individual genes or gene groups (e.g., **Figures 2C, D**).

Based on pairwise comparisons between fly conditions, DEGs were identified using the criteria: an adjusted p-value < 0.05 and the fold change > 2 or < 1/2 (**Table S2**). In NP3253>Kir2.1 flies, expression levels of 408 and 345 genes (under sucrose- and *Pe*-feeding conditions, respectively) were upregulated and 217 and 408 were downregulated, compared to those in the control. In NP3253>dTrpA1 flies, expression levels of 83 and 103 genes (under sucrose- and *Pe*-feeding conditions, respectively) were upregulated and 149 and 130 were downregulated, compared to those in the control. Consistent with the MDS analysis, inactivation of NP3253 neurons altered the expression of more genes than neuronal activation.

Upon comparing the sucrose- and *Pe*-feeding conditions, DEGs were detected as 3, 97, and 100 genes in the control, NP3253>Kir2.1, and NP3253>dTrpA1 flies, respectively. These results suggest that *Pe*-feeding altered the expression levels of a larger number of genes under the activation or inactivation of NP3253 neurons compared to those in the control flies. Finally, we identified 1139 DEGs in total from all pairwise comparisons.

### Some of the DEG groups showed enrichments of immune- and redox-related genes

To characterize gene expression patterns, we calculated the normalized variations (Z-scores) between fly conditions for the 1139 DEGs. Based on the heatmap of Z-scores, the DEGs were categorized into 12 cluster groups (**Figure 2B**, color bars with group numbers). Group 1 included 353 genes with upregulated expressions in NP3253>Kir2.1 flies compared to those in the other genotypes (**Figure S1** **;** **Figure 2C** shows a subset of these genes). Group 7 included 98 genes with downregulated expression in NP3253>Kir2.1 flies compared to those in the other genotypes (**Figure S1** **;** **Figure 2D** shows a subset of them).

The other 10 groups showed different expression patterns (**Figure S1**; see Discussion). Among the 12 cluster groups, we focused on groups 1 and 7 for the following analyses because of significant changes in gene expression in NP3253>Kir2.1 flies.

As noted in Introduction, NP3253-Gal4 flies expresses Gal4 in neural and non-neural cells. Therefore, the DEGs identified above may include genes that are unrelated to NP3253 neuronal functions. To exclude such genes, we selected DEGs from each cluster group whose expression in NP3253>Kir2.1 flies was rescued by elav-Gal80. We extracted 203 and 28 genes from groups 1 and 7 and termed them groups 1-2 and 7-2, respectively (**Figures 2C, D**, **S2**). Thus, the expression of these genes is regulated by NP3253-Gal4 positive neural cells.

Next, we performed GO analysis to examine the functions of the genes in each group. The 203 genes with upregulated expression levels in NP3253>Kir2.1 flies (group 1-2: **Data S2**) were enriched in the GO terms of innate immunity (in the BP category) (**Figure 2E**) and include the genes encoding AMPs and Toll signaling factors. We observed that the expression levels of these genes (e.g., *AttB*, *AttA*, *Dro*, and *CecA1* genes) were upregulated in NP3253>Kir2.1, with or without *Pe* infection (**Figure S3**). This suggests that the NP3253>Kir2.1 flies have a persistent inflammatory state in the intestine.

The 28 genes with downregulated expression levels in NP3253>Kir2.1 flies (group 7-2: **Data S3**) were enriched in the oxidoreductase and monooxygenase GO terms (in the MF category) (**Figure 2F**). These genes include the cytochrome P450 (Cyp) and glutathione S-transferase (GST) families, which are related to redox responses in cells. Previous studies have shown that expression levels of redox-related genes were upregulated during *Pe* oral infection in adult *Drosophila* (28). We observed that expression levels of these genes (*Cyp313b1*, *Cyp6d5*, and *Sodh1*) were downregulated in NP3253>Kir2.1 flies under sucrose- and *Pe*-feeding conditions (**Figure S3**), suggesting that the redox response of the intestine may be impaired in NP3253>Kir2.1 flies.

### Inactivation of NP3253 neurons increased susceptibility to commensal bacteria and ROS

**Figure 3:**
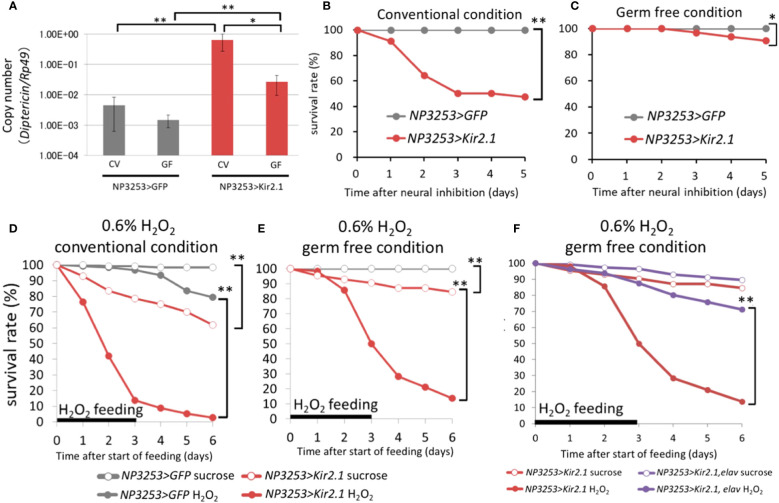
Effects of microbiota on gene expression and survival **(A)** RT-qPCR analysis for *DiptericinA* gene (*rp49* gene as internal control). The NP3253>GFP (gray) and NP3253>Kir2.1 (red) flies were reared under CV or GF conditions. Asterisks indicate statistically significant differences (*, p-value < 0.05; **, p-value < 0.01;, Wilcoxon rank sum tests). **(B, C)** Survival curves of NP3253>GFP and NP3253>Kir2.1 flies under CV **(B)** or GF **(C)** conditions. Asterisks indicate statistically significance (*, p-value < 0.05; **, p-value < 0.01) in the Log-rank test. Numbers of flies used in these experiments are **(B)** 55, 69, **(C)** 65, and 63 flies/3 vials (NP3253>GFP and NP3253>Kir2.1 flies, respectively). **(D, E)** Survival curves of NP3253>GFP and NP3253>Kir2.1 flies reared with 0.6% H_2_O_2_ under CV **(D)** and GF **(E)** conditions. Asterisks indicate statistically significance (p-value < 0.01) in the Log-rank test. Numbers of flies used in these experiments are **(D)** 120, 115, 121,117 flies/6 vials and **(E)** 117, 80, 110, 116 flies/4-6 vials (H_2_O_2_- or sucrose-fed NP3253>GFP flies and H_2_O_2_- or sucrose-fed NP3253>Kir2.1 flies, respectively). **(F)** Survival curves of NP3253>Kir2.1 (same as the data in E) and NP3253>Kir2.1, elav-Gal80 flies reared with 0.6% H_2_O_2_ under GF conditions. Asterisks indicate statistical significance (p-value < 0.05) in the Log-rank test. Numbers of flies used in these experiments are 111, 116 flies/6 vials (H_2_O_2_- or sucrose-fed NP3253>Kir2.1, elav-Gal80 flies).

Transcriptome analysis revealed that the immune reaction in the gut was activated in NP3253>Kir2.1 flies even without pathogen infection (group 1-2). We hypothesized that immune activation in NP3253>Kir2.1 flies might be induced by commensal bacteria in the gut, which are usually harmless in flies. To evaluate this hypothesis, we prepared GF axenic flies. First, we measured the expression levels of *DptA*, which encodes an AMP, under CV and GF conditions. Our RT-qPCR analysis showed that the expression of the *DptA* gene under CV conditions was increased in NP3253>Kir2.1 flies compared to that in the control flies; this increased expression was significantly suppressed by GF conditions (**Figure 3A**). In the control flies, there was no significant difference in *DptA* expression between the CV and GF conditions. Next, we performed survival assays and found that the survival rate of NP3253>Kir2.1 flies decreased to approximately 50% for 5 days, even without pathogen infection under CV conditions; however, it was restored under GF conditions (**Figures 3B, C**). These results suggest that NP3253>Kir2.1 flies are susceptible to microbiota and induce immune activation in the gut in a microbiota-dependent manner.

Furthermore, transcriptome analysis revealed that expression levels of certain genes related to redox reactions were downregulated in NP3253>Kir2.1 flies (group 7-2). We hypothesized that ROS sensitivity may be elevated in NP3253>Kir2.1 flies. To test this possibility, we measured the survival rate of NP3253>Kir2.1 flies under ROS stress conditions. NP3253>Kir2.1 flies treated with H_2_O_2_ showed a lower survival rate than the control flies under CV conditions (**Figure 3D**). Subsequently, we performed the same assay under GF conditions and found that NP3253>Kir2.1 flies were still vulnerable to H_2_O_2_ (**Figure 3E**). Thus, the ROS sensitivity of NP3253>Kir2.1 flies was independent of the gut commensal bacteria. In addition, we observed that ROS sensitivity in NP3253>Kir2.1 flies was rescued by elav-Gal80 (**Figure 3F**). These results indicate that NP3253 neurons regulate ROS sensitivity in the gut in a microbiota-independent manner.

### Transcriptional profiling associated with the activity of NP3253 neurons under GF conditions

Immune- and redox-related genes were identified as DEGs between NP3253>Kir2.1 and control flies in the RNA-seq analysis (EXP1). Next, we investigated whether the expression of these genes was microbiota-dependent and performed another RNA-seq analysis of the gut using axenic flies (referred to as EXP2). Samples were prepared under the following four fly conditions (genotypes + rearing conditions): control (NP3253>GFP) and neuron-inactivated (NP3253>Kir2.1) flies reared under CV and GF conditions. Total RNAs were extracted from guts and subjected to RNA-Seq analysis (**Table S3**, **Data S4**).

**Figure 4:**
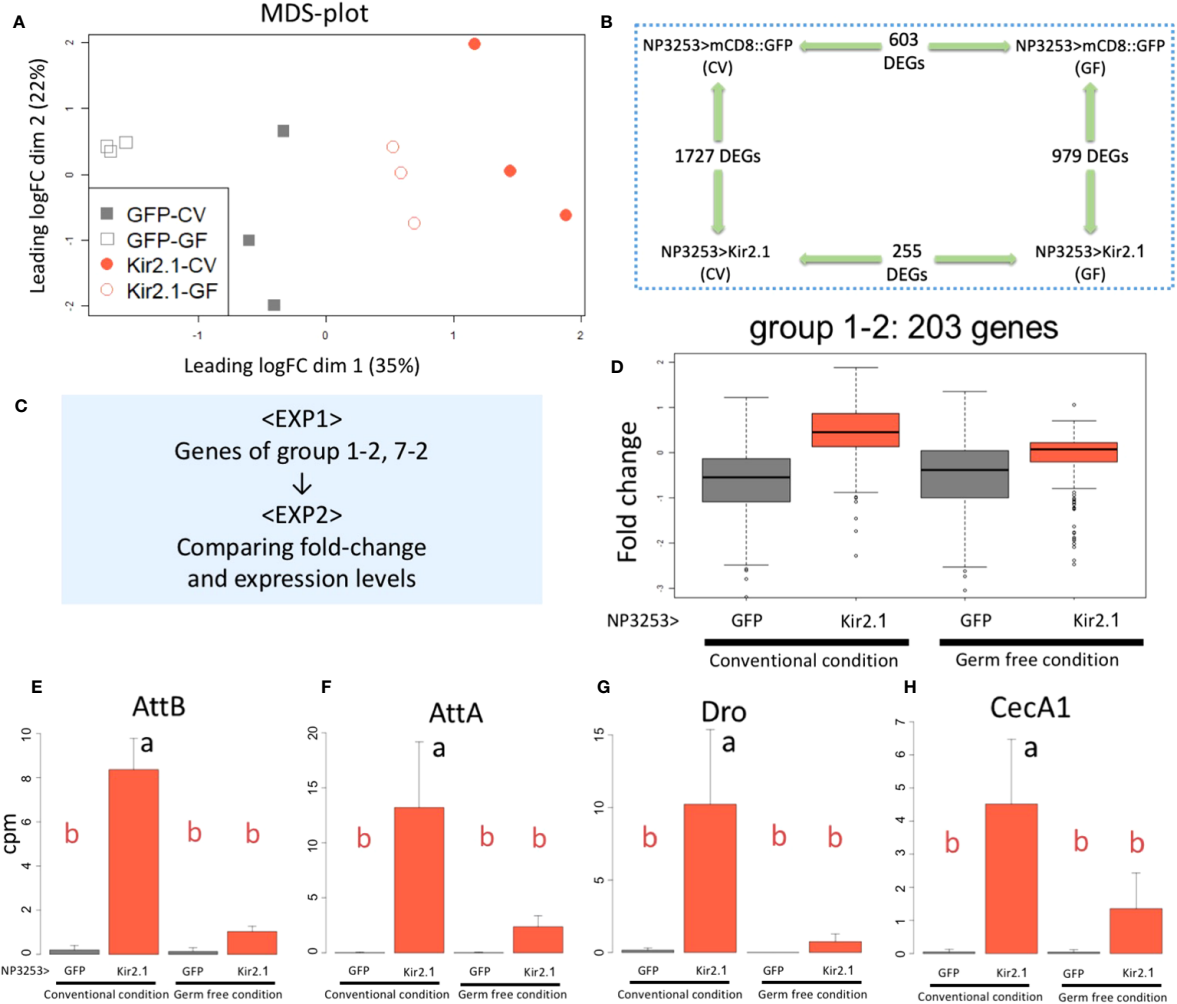
RNA-seq analysis under GF conditions (EXP2) **(A)** MDS plots of transcriptomes for all samples (EXP2). NP3253>GFP (gray) and NP3253>Kir2.1 (red) flies were reared under CV or GF conditions. **(B)** DEGs detected using pairwise comparisons between two conditions. **(C)** Strategy for integrated analysis of EXP1 and EXP2. **(D)** Box plots of fold-changes from the averages of the EXP2 data for 203 genes categorized into group 1-2 in the EXP1. **(E-H)** Bar plots of RNA-seq data (cpm) from the EXP2 for AMP genes: *AttB* **(E)***, AttA***(F)***, Dro* **(G)**, and *CecA1* **(H)**. Different alphabets indicate statistically significant (p-value < 0.05) differences between the conditions assessed using one-way ANOVA and Tukey HSD *post-hoc* tests.

The MDS plot of EXP2 showed that the overall gene expression patterns were similar between replicate samples under the same fly conditions. The GF-reared samples showed less variation between replicates than the CV-reared samples for both genotypes (**Figure 4A**). We performed pairwise comparisons between genotypes and rearing conditions and identified DEGs using the criteria same as those used for EXP1. Comparisons between genotypes (NP3253>Kir2.1 vs. control flies) identified 1,727 and 979 genes as DEGs under CV and GF conditions, respectively (**Figure 4B**). Comparisons between the CV and GF conditions identified 255 and 603 genes as DEGs in NP3253>Kir2.1 and control flies, respectively. These results suggest that gene expression in the gut is considerably altered in response to commensal gut bacteria. This change is lesser in NP3253>Kir2.1 flies than in control flies, suggesting that NP3253 neurons may contribute to responses of the gut to commensal bacteria.

### Expression of some AMP genes was upregulated by microbiota in NP3253>Kir2.1 flies

To determine whether the DEGs identified in EXP1 were responsive to commensal bacteria, we analyzed the EXP2 data for the genes of each cluster group of EXP1 (**Figure 4C**). As seen in EXP1 (**Figure 2C**), 203 genes of group 1-2 showed higher expression in NP3253>Kir2.1 flies than in control flies, under CV conditions in EXP2 (**Figure 4D**). However, under GF conditions, their expression in NP3253>Kir2.1 flies was not significantly different from that in control flies. As immune-related genes were mostly enriched in this group, we observed the expression patterns of some AMP genes. Consequently, expression levels of these genes (e.g., *AttB*, *AttA*, *Dro*, and *CecA1*) were upregulated in CV-reared NP3253>Kir2.1 flies, and this upregulation was suppressed in GF-reared flies (**Figures 4E-H**). These results suggest that the inactivation of NP3253 neurons enhances the immune responses to gut commensal bacteria.

### Certain redox-related genes contribute to defense against oral bacterial infection

**Figure 5:**
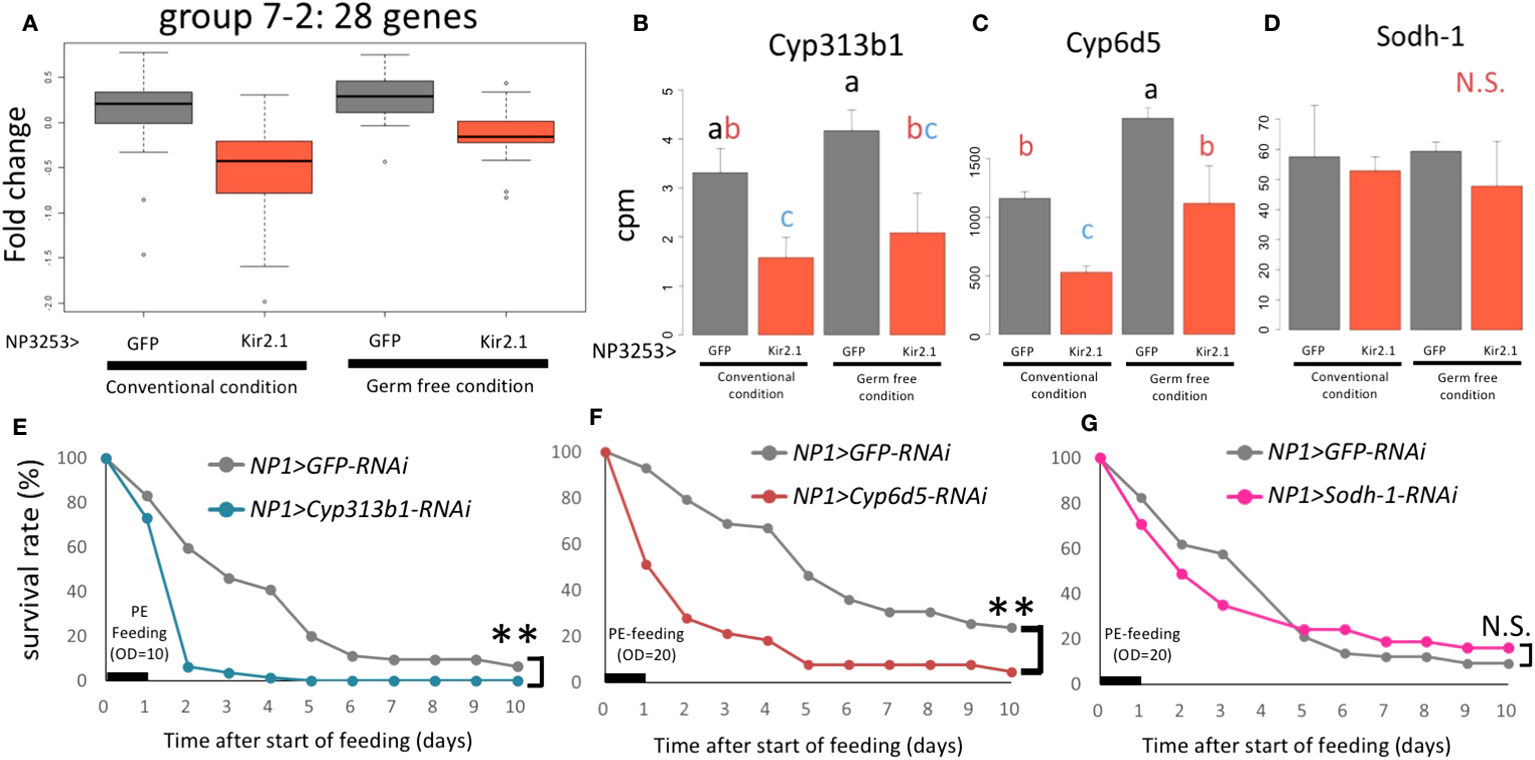
**The expression of redox-related genes under the control of NP3253 neurons **(A)**** Box plots of fold-changes from the averages of the EXP2 data for 28 genes categorized into group 7-2 in the EXP1. **(B-D)** Bar plots of RNA-seq data (cpm) for redox-related genes: *Cyp313b1* **(B)**, *Cyp6d5* **(C)**, and *Sodh-1* **(D)**. Different alphabets indicate statistically significant (p-value < 0.05) differences between the conditions assessed using one-way ANOVA and Tukey HSD *post-hoc* tests. N.S. indicates non-significance. **(E-G)** Survival assays under *Pe* oral infection (OD = 10 **(E)**, 20 **(F, G)**) for RNAi lines: NP1>Cyp313b1RNAi **(E)**, Cyp6d5-RNAi **(F)**, and Sodh-1-RNAi **(G)**. NP1>GFP RNAi was used as controls. Numbers of flies used in these experiments were **(E)** 60, 62, **(F)** 58, 63, **(G)** 68, and 71 flies/3 vials (control and knockdown lines, respectively). Asterisks and N.S. indicate statistical significance (p-value < 0.01) and non-significance in the Log-rank test, respectively.

In EXP1, 28 genes in group 7-2 showed lower expression in NP3253>Kir2.1 flies than in the control (**Figure 2D**). In EXP2, similar expression patterns were observed for these genes under CV and GF conditions (**Figure 5A**). This result suggests that this group of genes related to redox reactions is regulated by NP3253 neurons independent of the gut microbiota. For example, Cyp6d5 and Cyp313b1 expression was downregulated in NP3253>Kir2.1 flies compared to that in the control flies, under CV and GF conditions (**Figures 5B, C**). Sodh-1 was listed as a DEG in EXP1 (**Figure S3**) but not in EXP2 (**Figure 5D**), probably because of large variance in the data.

We hypothesized that some redox-related genes contribute to gut immunity during pathogenic infections. To test this hypothesis, we measured the survival rates of knockdown flies after *Pe* infection. For the knockdown experiments, we used the NP1-Gal4 line, which induces RNAi for each gene in intestinal epithelial cells (29). The results showed that knockdown of *Cyp6d5* and *Cyp313b1* reduced the survival rate after *Pe* infection (**Figures 5E, F**), while under sucrose-feeding conditions, these flies were almost as alive as the control flies (NP1>GFP RNAi; **Figure S4**). In contrast, knockdown of the *Sodh-1* did not affect survival rates after *Pe* infection (**Figure 5G**), suggesting that a subset of redox-related genes contributes to *Pe* susceptibility. We suggest that NP3253 neurons directly regulate the expression of redox-related genes in the gut, essential for defense against oral pathogenic infections.

## Discussion

This study investigated the role of NP3253 neurons in pathogenic oral infections. We showed that hyperactivation of NP3253 neurons increased the survival rate of flies after *Pe* infection but did not alter the bacterial load in the body. This finding suggests that activation of NP3253 neurons increases tolerance to pathogenic infections. Two types of immune defense operate in the gut: resistance, which eliminates pathogens by killing or removing them, and tolerance, which protects the host from damage caused by infection (12). NP3253 neurons may be involved in both types of defenses, as their inactivation increases the *Pe* load in the body. In a previous study (18), we observed a leaky gut phenotype in NP3253>Kir2.1 flies, suggesting that the increased *Pe* load in NP3253>Kir2.1 flies might be due to bacterial leakage from the gut. However, this model does not fully explain the phenotypes because the leaky gut phenotype was only observed in a fraction of individuals (10-20%), whereas an increased *Pe* load was detected in all individuals examined (**Figure 1E**; each dot represents data from one individual). Thus, we propose that NP3253 neurons may regulate resistance and tolerance to oral pathogen infections.

RNA-seq analysis revealed a small difference between the control and hyperactivated NP3253 neurons (NP3253>dTrpA1 flies) but a large difference between the control and inactivated NP3253 neurons (NP3253>Kir2.1 flies), thus suggesting that NP3253 neurons may be partially activated even in control flies. Currently, we do not know which factors (molecules or environments) activate NP3253 neurons. NP3253 neurons are localized in the subesophageal ganglion of the brain, where many gustatory neurons are innervated (30). We speculate that NP3253 neurons may be involved in sensing the chemical environment of the gut (e.g., nutrients and pathogens). To address the physiological functions of NP3253 neurons, it is necessary to identify the factors that activate them (**Figure S5**).

Using EXP1, we identified the DEGs and categorized them into 12 cluster groups. Although we focused on groups 1 and 7 in this study, the other 10 groups showed diverse expression patterns (**Figure S1**). For example, 244 genes in group 4 showed expression patterns similar to those in group 7; however, their expression was relatively low in NP3253>dTrpA1 flies. We note that group 4 included some GST and Cyp genes, similar to group 7. These genes might contribute to redox response during oral infection. Expression levels of 44 genes in group 5 were upregulated in NP3253>dTrpA1 flies compared to those in other genotypes. Some of these genes may be involved in tolerance to infection upon activation of NP3253 neurons. Thirty-three genes in group 12 showed *Pe*-responsive expression in control and NP3253>dTrpA1 flies but not in NP3253>Kir2.1 flies. Furthermore, we identified DEGs under GF conditions from another RNA-seq analysis (EXP2), although we did not perform the cluster analysis of DEGs. Thus, some of these DEGs may play critical roles in brain-gut-microbiota interactions. However, further studies are required to elucidate their roles.

Our transcriptome analyses revealed that the expression of immune-related genes was upregulated in NP3253>Kir2.1 flies, even in the absence of pathogenic infection, and that the expression of these genes was significantly suppressed under GF conditions. These results suggest that the expression of immune-related genes in NP3253>Kir2.1 flies depends on the presence of the microbiota. The guts of NP3253>Kir2.1 flies may be hypersensitive to bacteria or their derived components (**Figure S5**). Consistently, even under GF conditions, *DptA* expression was slightly but significantly upregulated in NP3253>Kir2.1 flies compared to that in the control (**Figure 3A**). Alternatively but not exclusively, gut commensal bacteria could be overgrowing in NP3253>Kir2.1 flies, similar to the observation that the number of *Pe* bacteria increased in these flies (**Figure 1E**). In either case, the gut of NP3253>Kir2.1 flies would be highly inflammatory, thereby reducing their survival rate. Thus, we suggest that in normal conditions, NP3253 neurons may suppress immune activation triggered by microbiota (**Figure S5**). In *Drosophila*, commensal gut bacteria play beneficial roles by providing the host nutrients (e.g., short-chain fatty acids) and altering the immune response (11). However, when the abundance and composition of commensal microbiota change, as in old flies, they become harmful to the host (31). We hypothesize that NP3253 neurons may help maintain the appropriate abundance and composition of the gut microbiota in healthy flies (**Figure S5**).

Furthermore, transcriptome analysis revealed that the expression of redox-related genes decreased in NP3253>Kir2.1 flies, independent of the gut commensal bacteria. This finding suggests that NP3253 neurons directly regulate the expression of redox-related genes in the gut (**Figure S5**). Expression levels of certain redox-related enzymes (e.g., GSTs) are upregulated in the gut following bacterial infection and oxidant exposure (32, 33). Our knockdown experiments showed that at least two enzymes, Cyp6d5 and Cyp313b, contribute to survival after *Pe* infection. A previous study showed that Cyp6d5 expression is upregulated upon caffeine ingestion and regulates caffeine metabolism in flies, suggesting that Cyp6d5 contributes to xenobiotic detoxification (34). The susceptibility of NP3253>Kir2.1 flies to oral infection may be due to the toxicity of pathogen-derived xenobiotics. Additionally, GSTs and oxidoreductases are believed to regulate ROS levels (35, 36). In the *Drosophila* gut, ROS are produced in intestinal epithelial cells in response to the amount of uracil secreted by the bacteria. ROS kill bacteria and simultaneously damage the host epithelial cells (12, 13). Intestinal stem cells (ISCs) rapidly divide and differentiate to repair the damage. In NP3253>Kir2.1 flies, upd3-dependent ISC division was enhanced in the anterior midgut (18), possibly due to excessive ROS-induced damage. NP3253>Kir2.1 flies showed reduced survival rates after H_2_O_2_ feeding, even under axenic conditions, suggesting that their ROS sensitivity is high regardless of the microbiota. This finding suggests that NP3253 neurons may regulate productivity and sensitivity to ROS via the expression of redox-related genes. To test this hypothesis, we examined ROS levels and cellular responses to ROS in different fly lines.

The gut contains several cell types, including epithelial cells, ISC, and endocrine cells. As this study performed RNA-seq analysis using the whole gut, we did not determine which cell types DEGs are expressed in. Recent studies have provided detailed data from single-cell RNA-seq analyses of the whole body, gut, brain, and other tissues (37, 38). These databases are useful for assessing cell type-specific gene expression in the gut. The expression of immune-related genes is known to be upregulated in the intestinal epithelial and endocrine cells of the anterior midgut during an infection (29, 39). Thus, immune-related genes may be induced in the intestinal epithelial and endocrine cells of NP3253>Kir2.1 flies. As the knockdown of *Cyp6d5* and *Cyp313b* using NP1-Gal4 shortened survival after *Pe* infection, we propose that these enzymes function in intestinal epithelial cells.

We propose that NP3253 neurons are required for sensing and regulating the physiological state of the gut. As NP3253 cells contain dozens of neurons and play multiple roles in gut physiology, whether all or only a subset of neurons are functional remains unclear. In addition, whether the same or different NP3253 neurons are involved in responses to pathogens, microbiota, and ROS remains unclear. In the future, we will need to individually analyze the subsets of NP3253 neurons and investigate their roles. This study describes the involvement of NP3253 neurons in the regulation of redox response and microbiota-triggered immune activation in the *Drosophila* gut (**Figure S5**). The pathogenesis of inflammatory bowel diseases (IBD), such as ulcerative colitis and Crohn’s disease, involves a breakdown in redox regulation and abnormal immune activation (40). Determining whether human neurons homologous to *Drosophila* NP3253 neurons are involved in gut homeostasis may provide clues for developing treatments for IBD. These studies would contribute to understanding brain-gut-microbiota interactions in healthy and disease states.

## Data availability statement

The datasets presented in this study can be found in online repositories. The names of the repository/repositories and accession number(s) can be found in the article/**Supplementary Material**.

## Ethics statement

Ethical review and approval was not required for this study in accordance with the local legislation and institutional requirements.

## Author contributions

NF: Conceptualization, Data curation, Formal Analysis, Funding acquisition, Investigation, Methodology, Project administration, Supervision, Validation, Writing – original draft, Writing – review & editing. HH: Data curation, Investigation, Methodology, Writing – original draft. KI: Data curation, Formal Analysis, Investigation, Methodology, Writing – original draft. TS: Data curation, Formal Analysis, Investigation, Methodology, Writing – original draft. Q-DN: Data curation, Formal Analysis, Investigation, Methodology, Writing – original draft. KF: Data curation, Formal Analysis, Investigation, Methodology, Writing – original draft. WI-O: Conceptualization, Project administration, Supervision, Validation, Writing – review & editing. HK: Conceptualization, Project administration, Supervision, Validation, Writing – review & editing. HT: Conceptualization, Project administration, Supervision, Validation, Writing – review & editing. SK: Conceptualization, Funding acquisition, Project administration, Supervision, Validation, Writing – review & editing.

## Supplementary material

Supplementary Figure 1 Boxplot for all cluster groups. Box plots of z-scores of DEGs categorized into cluster groups (EXP1). In this study, we focused on groups 1 and 7.

Supplementary Figure 2 Clustering for subgroups of groups 1 and 7. After adding data from elav-Gal80, NP3253>Kir2.1 flies, cluster analyses were performed for groups 1 and 7. Subsequently, group 1 was divided into subgroups 1-1 and 1-2 (352 genes in total; one gene was removed owing to low average reads). Group 7 was divided into subgroups 7-1, 7-2, and 7-3. Box plots of the z-scores of the DEGs were categorized into each subgroup. We focused on groups 1-2 and 7-2 (same as **Figures 2C, D**) for further analysis because gene expression in NP3253>Kir2.1 was rescued by elav-Gal80.

Supplementary Figure 3 Expression of some of the immune- and redox-related genes (EXP1). Bar plots of RNA-seq data (cpm) for immune-related (*AttB, AttA, Dro*, and *CecA1*) and redox-related (*Cyp313b1, Cyp6d5*, and *Sodh1*) genes. Rescue by elav-Gal80 indicates that the phenotype is the results of Kir2.1 being expressed in neuronal cells rather than nonneuronal cells.

Supplementary Figure 4 Survival for the knockdown lines under sucrose-feeding conditions. Survival assays under sucrose-feeding conditions for RNAi lines: NP1> Cyp313b1-RNAi (left), Cyp6d5-RNAi (middle), and Sodh-1-RNAi (right). NP1 > GFP RNAi was used as a control. The numbers of flies used in these experiments were (left) 125 and 102 flies/6 vials; (middle) 54 and 48 flies/3 vials; and (right) 64 and 42 flies/2-3 vials (control and knockdown lines, respectively).

Supplementary Figure 5 Hypothetical model for roles of NP3253 neurons. In our model, NP 3253 neurons may suppress the expression of immune-related genes that are otherwise induced by microbiota. NP3253 neurons may also regulate the abundance of microbiota and/or the immune sensitivity against microbiota. Moreover, NP3253 neurons may directly regulate the expression of redox-related genes in the gut. Thus, NP3253 neurons may organize the immune and redox responses in the gut. It is unknown which factors (molecules or environments) activate NP3253 neurons.

Supplementary Table 1 Summary of the RNA-seq analysis (EXP1). Sample name (genotype, feeding condition, replica number), label (common for Data S1 and DRA008209), total reads, mapped reads, and mapping rates for each sample of RNA-seq analysis (EXP1) are indicated.

Supplementary Table 2 Identification of DEGs from pairwise comparisons (EXP1). DEGs were identified in each pairwise comparison. The number of upregulated and downregulated genes was determined.

Supplementary Table 3 Summary of the RNA-seq analysis (EXP2). Sample name (genotype, rearing condition, replica number), label (common for Data S4 and DRA012434), total reads, mapped reads, and mapping rates for each sample of RNAseq analysis (EXP2) are indicated.

Supplementary Data Sheet 1 RNA-seq data (EXP1). Normalized count data (cpm) of RNA-seq (EXP1) for all genes (Flybase Gene ID) and all samples (label in **Table S1**).

Supplementary Data Sheet 2 List of the group 1-2 genes. Flybase Gene ID on RNA-seq data (#SUBMITTED), Flybase Gene ID on Flybase (FB2023_03) (FBID_KEY), GO terms (Biological Process), GO terms (Cellular Component), GO terms (Molecular Function) and gene symbol are listed for group 1-2 genes.

Supplementary Data Sheet 3 List of the group 7-2 genes. Flybase Gene ID on RNA-seq data (#SUBMITTED), Flybase Gene ID on Flybase (FB2023_03) (FBID_KEY), GO terms (Biological Process), GO terms (Cellular Component), GO terms (Molecular Function) and gene symbol are listed for group 7-2 genes.

Supplementary Data Sheet 4 RNA-seq data (EXP2). Normalized count data (cpm) of RNA-seq (EXP2) for all genes (Flybase Gene ID) and all samples (label in **Table S3**).
