# Supplementary figures and images for: Neural control of redox response and microbiota-triggered inflammation in Drosophila gut
Source: Front Immunol. 2023 Oct 26;14:1268611. doi: 10.3389/fimmu.2023.1268611 (PMC10642236; doi:10.3389/fimmu.2023.1268611)

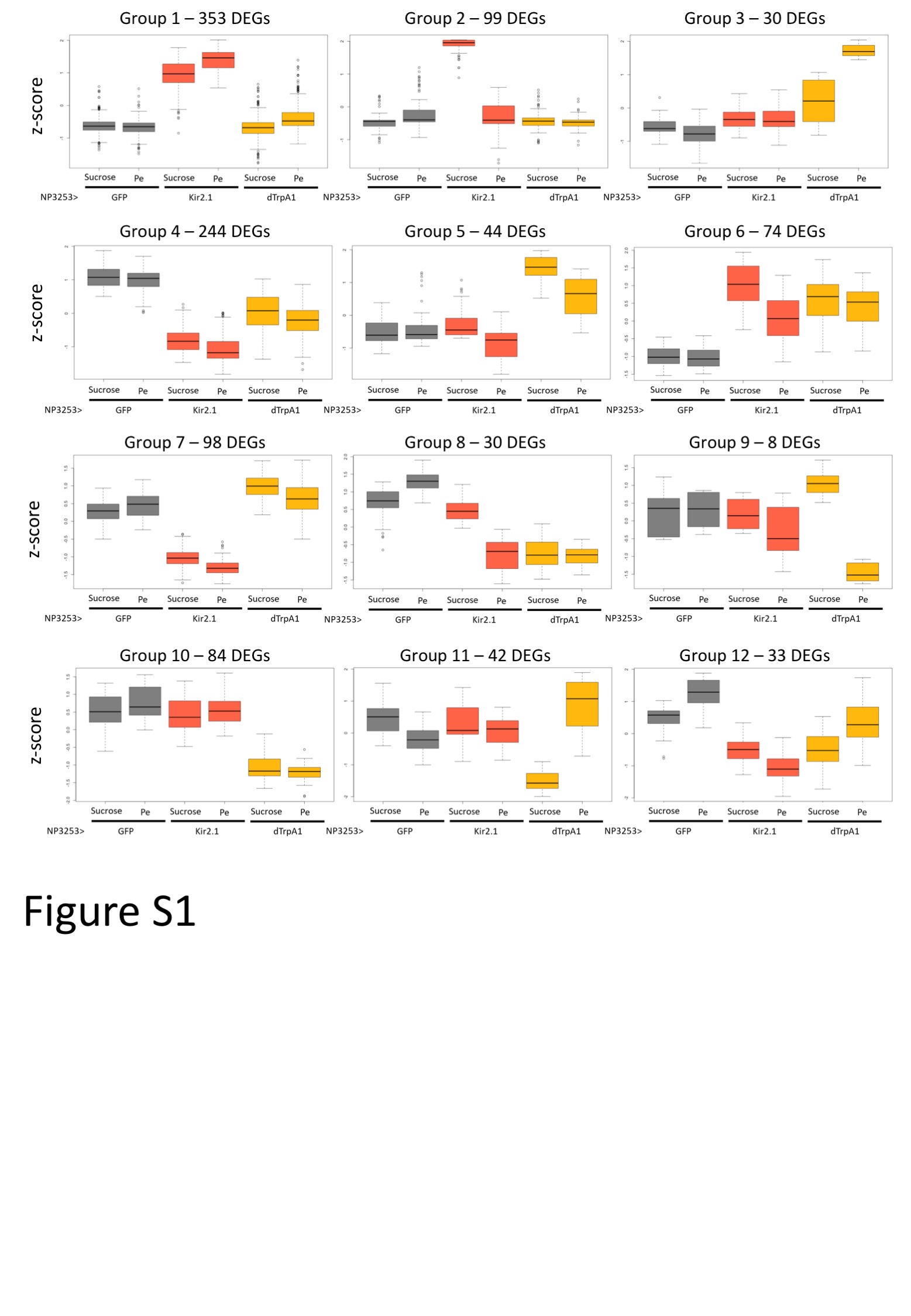

Supplement: Supplementary Figure 1 — Boxplot for all cluster groups. Box plots of z-scores of DEGs categorized into cluster groups (EXP1). In this study, we focused on groups 1 and 7. [file Image_1.jpeg]

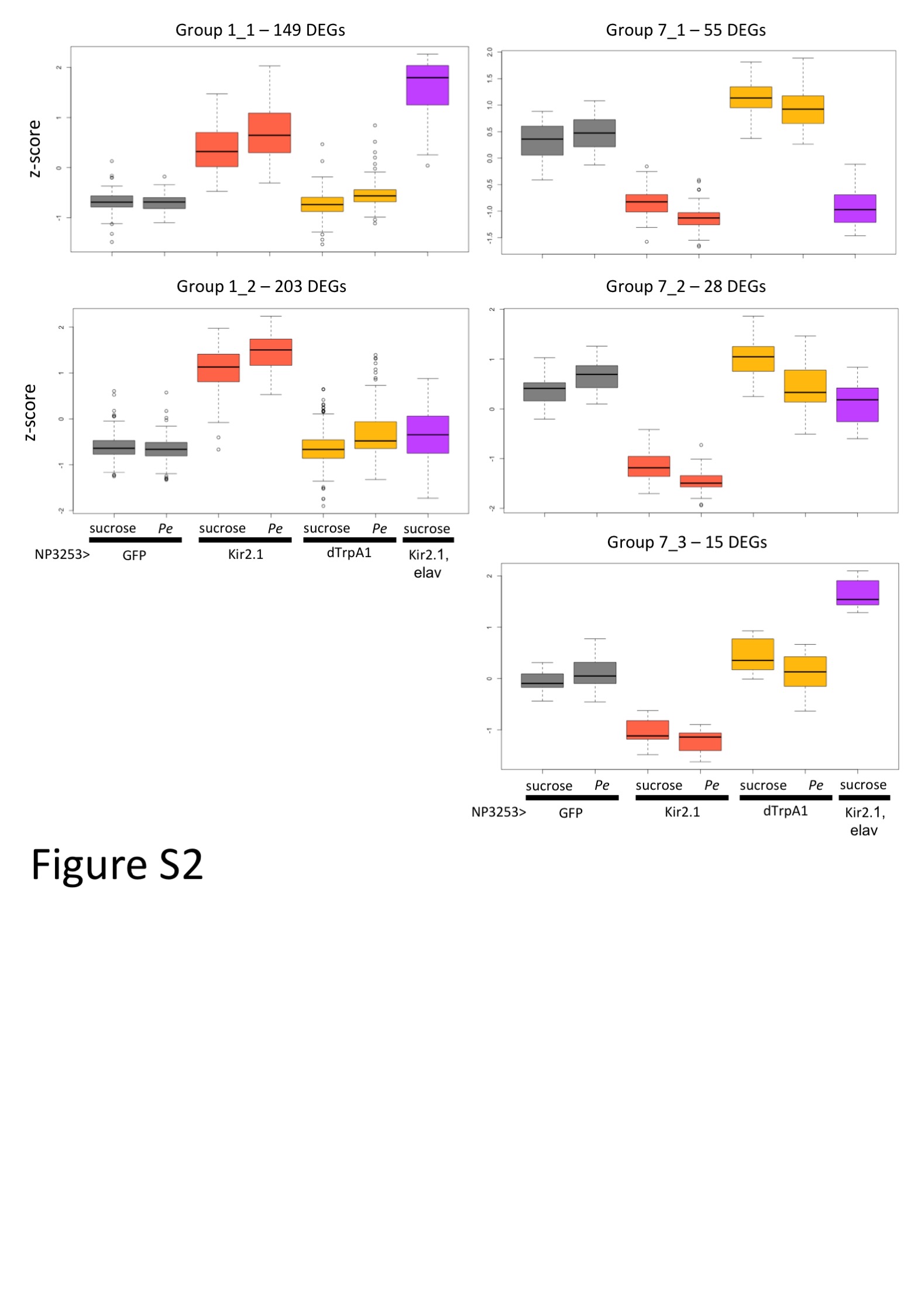

Supplement: Supplementary Figure 2 — Clustering for subgroups of groups 1 and 7. After adding data from elav-Gal80, NP3253>Kir2.1 flies, cluster analyses were performed for groups 1 and 7. Subsequently, group 1 was divided into subgroups 1-1 and 1-2 (352 genes in total; one gene was removed owing to low average reads). Group 7 was divided into subgroups 7-1, 7-2, and 7-3. Box plots of the z-scores of the DEGs were categorized into each subgroup. We focused on groups 1-2 and 7-2 (same as Figures 2C, D ) for further analysis because gene expression in NP3253>Kir2.1 was rescued by elav-Gal80. [file Image_2.jpeg]

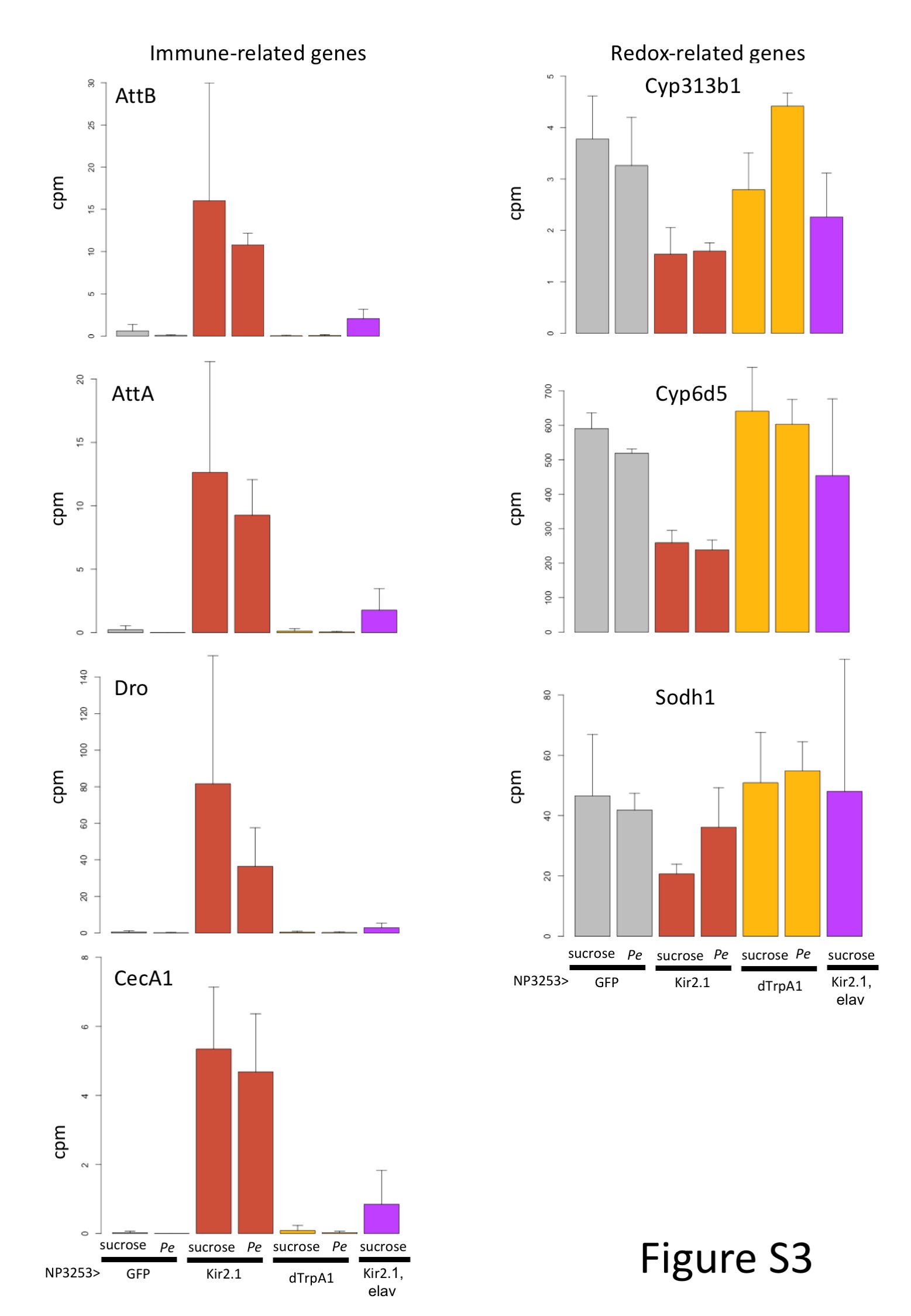

Supplement: Supplementary Figure 3 — Expression of some of the immune- and redox-related genes (EXP1). Bar plots of RNA-seq data (cpm) for immune-related (AttB, AttA, Dro, and CecA1) and redox-related (Cyp313b1, Cyp6d5, and Sodh1) genes. Rescue by elav-Gal80 indicates that the phenotype is the results of Kir2.1 being expressed in neuronal cells rather than nonneuronal cells. [file Image_3.jpeg]

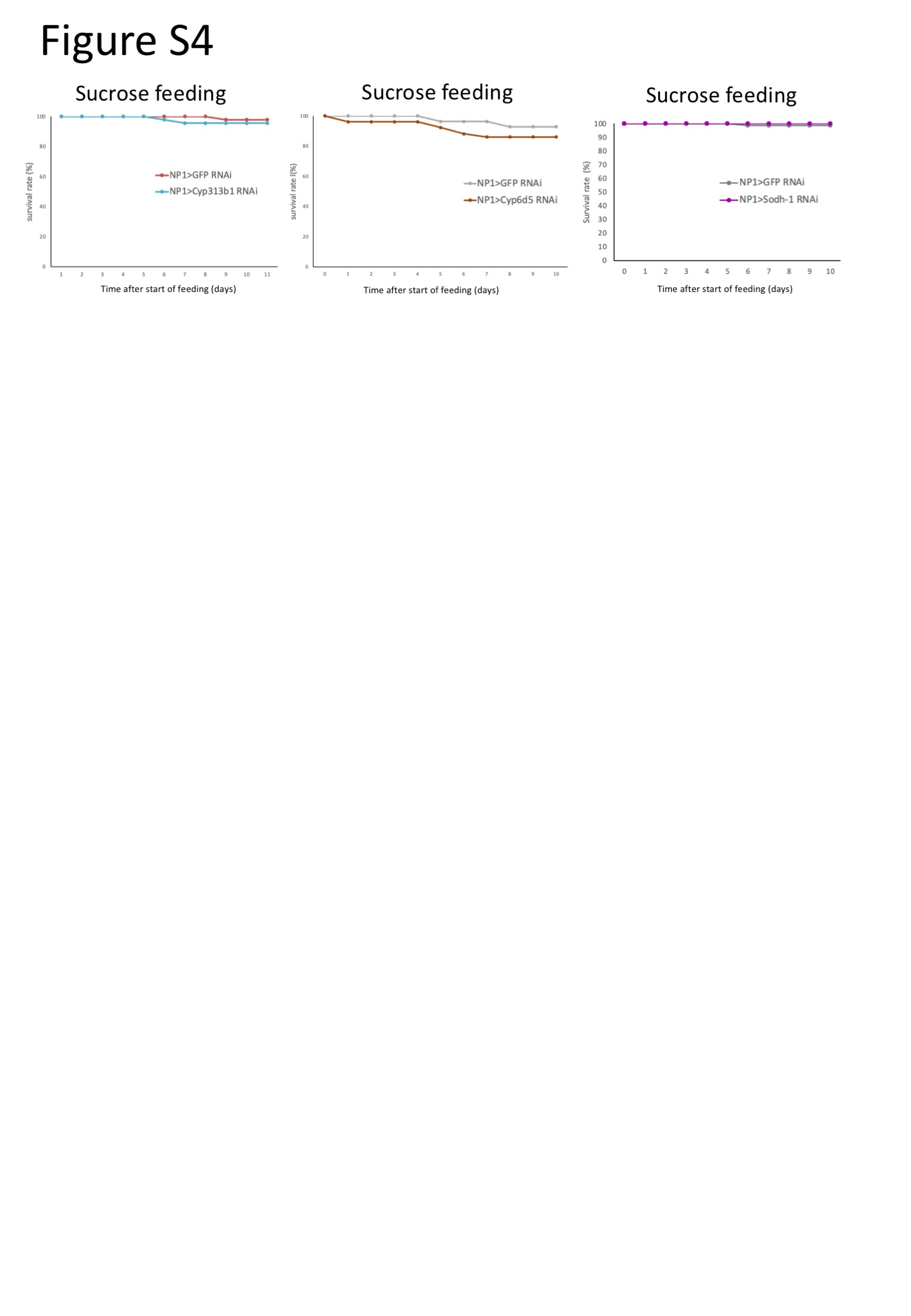

Supplement: Supplementary Figure 4 — Survival for the knockdown lines under sucrose-feeding conditions. Survival assays under sucrose-feeding conditions for RNAi lines: NP1> Cyp313b1-RNAi (left), Cyp6d5-RNAi (middle), and Sodh-1-RNAi (right). NP1 > GFP RNAi was used as a control. The numbers of flies used in these experiments were (left) 125 and 102 flies/6 vials; (middle) 54 and 48 flies/3 vials; and (right) 64 and 42 flies/2-3 vials (control and knockdown lines, respectively). [file Image_4.jpeg]

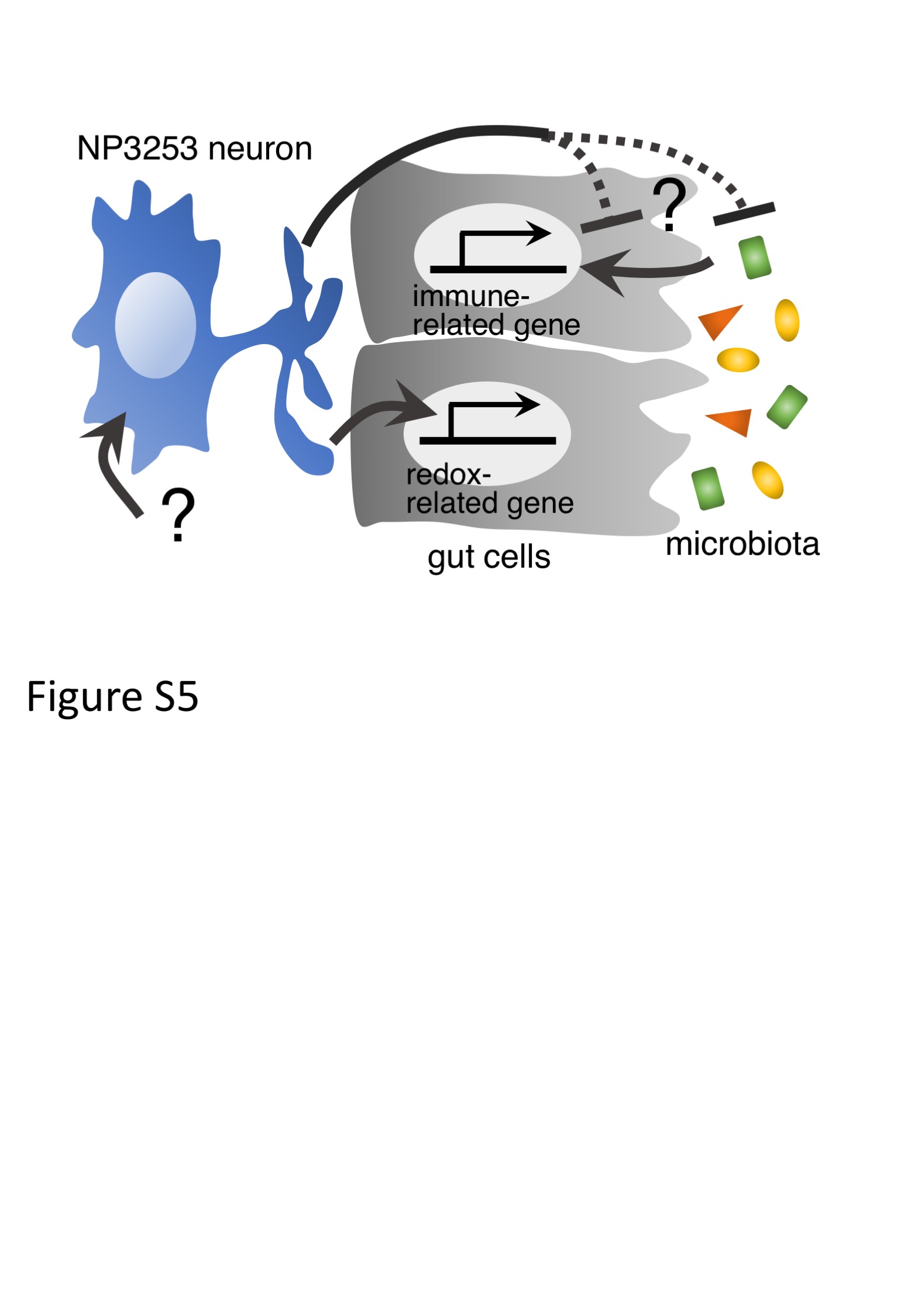

Supplement: Supplementary Figure 5 — Hypothetical model for roles of NP3253 neurons. In our model, NP 3253 neurons may suppress the expression of immune-related genes that are otherwise induced by microbiota. NP3253 neurons may also regulate the abundance of microbiota and/or the immune sensitivity against microbiota. Moreover, NP3253 neurons may directly regulate the expression of redox-related genes in the gut. Thus, NP3253 neurons may organize the immune and redox responses in the gut. It is unknown which factors (molecules or environments) activate NP3253 neurons. [file Image_5.jpeg]

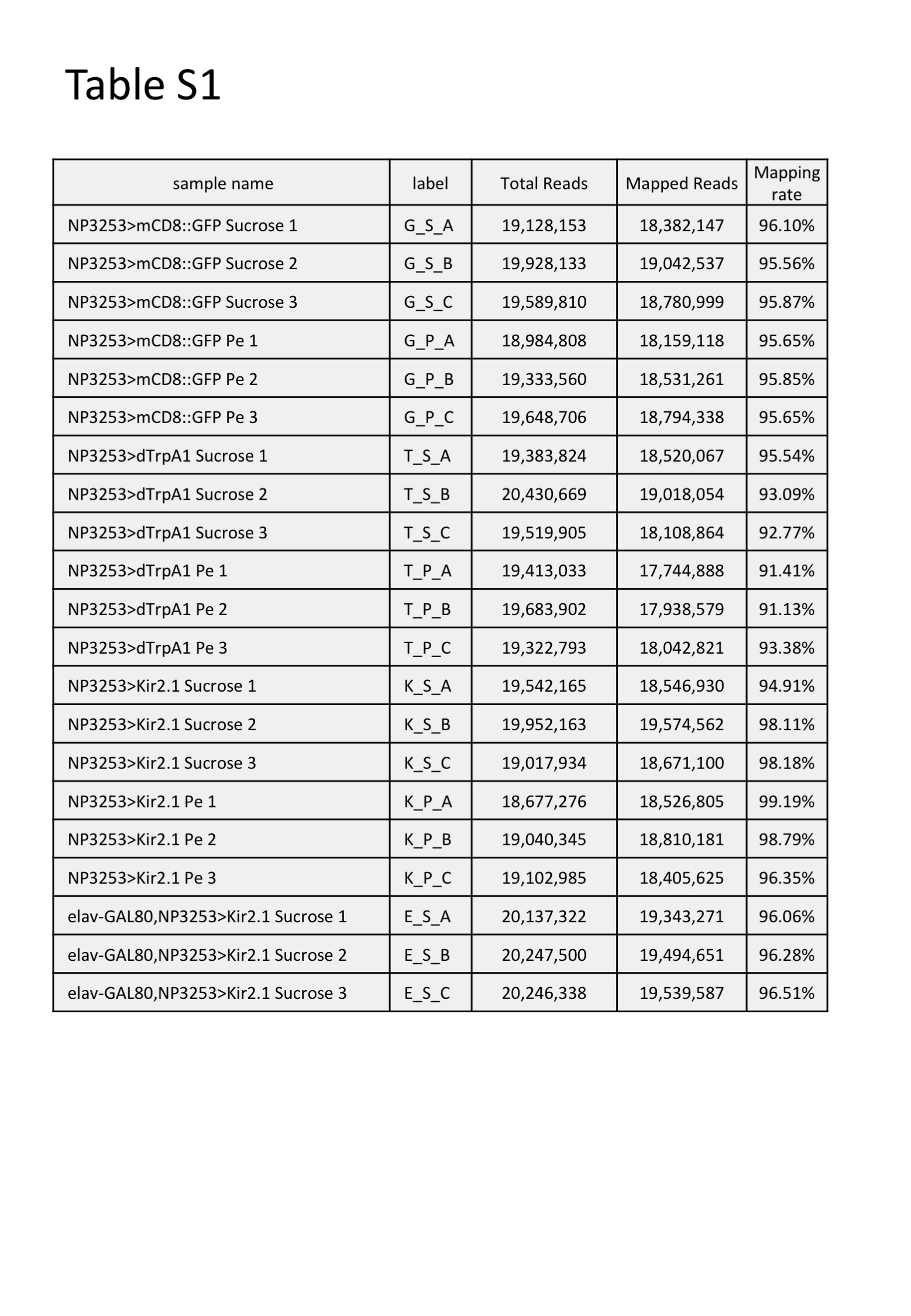

Supplement: Supplementary Table 1 — Summary of the RNA-seq analysis (EXP1). Sample name (genotype, feeding condition, replica number), label (common for Data S1 and DRA008209), total reads, mapped reads, and mapping rates for each sample of RNA-seq analysis (EXP1) are indicated. [file Image_6.jpeg]

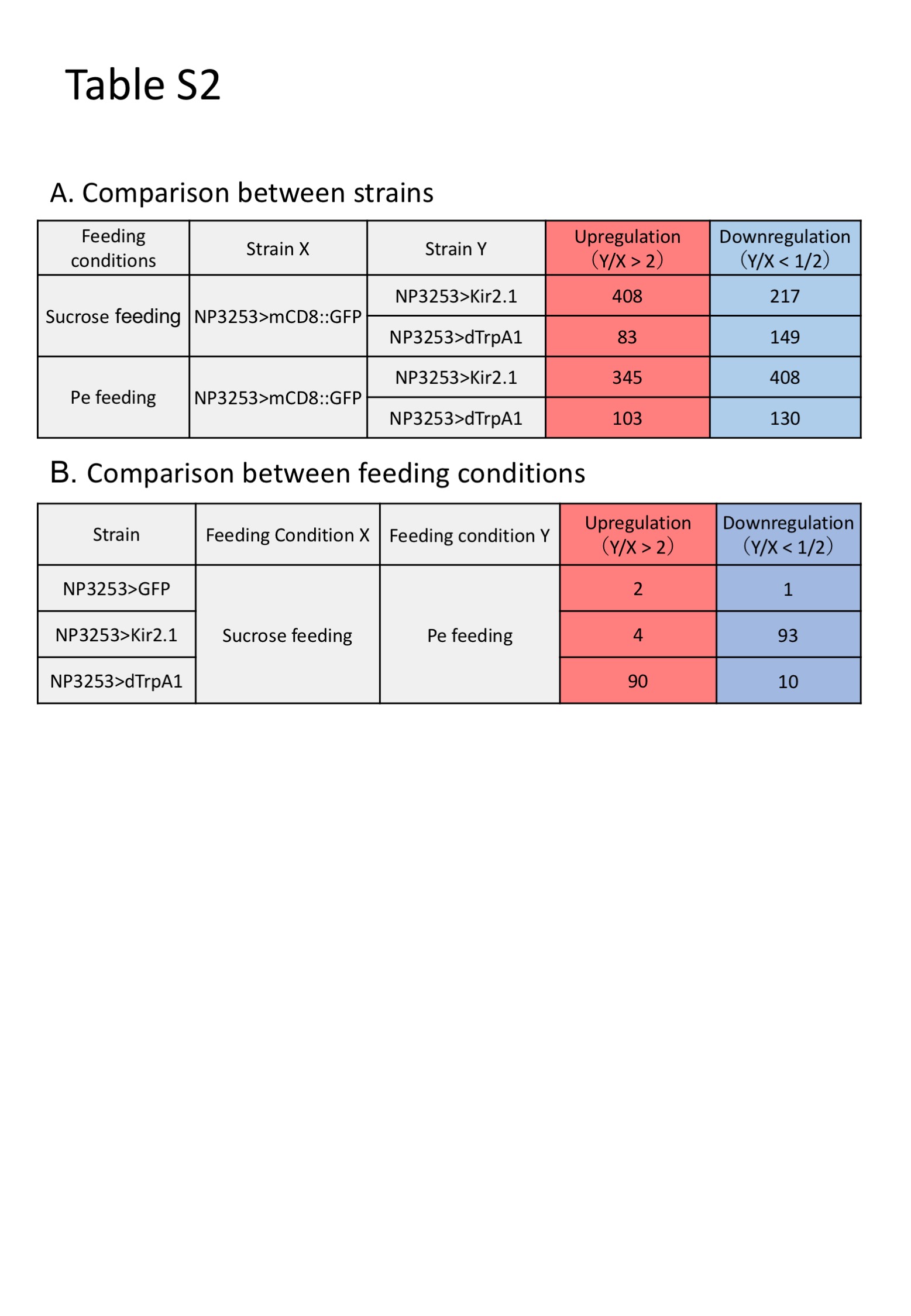

Supplement: Supplementary Table 2 — Identification of DEGs from pairwise comparisons (EXP1). DEGs were identified in each pairwise comparison. The number of upregulated and downregulated genes was determined. [file Image_7.jpeg]

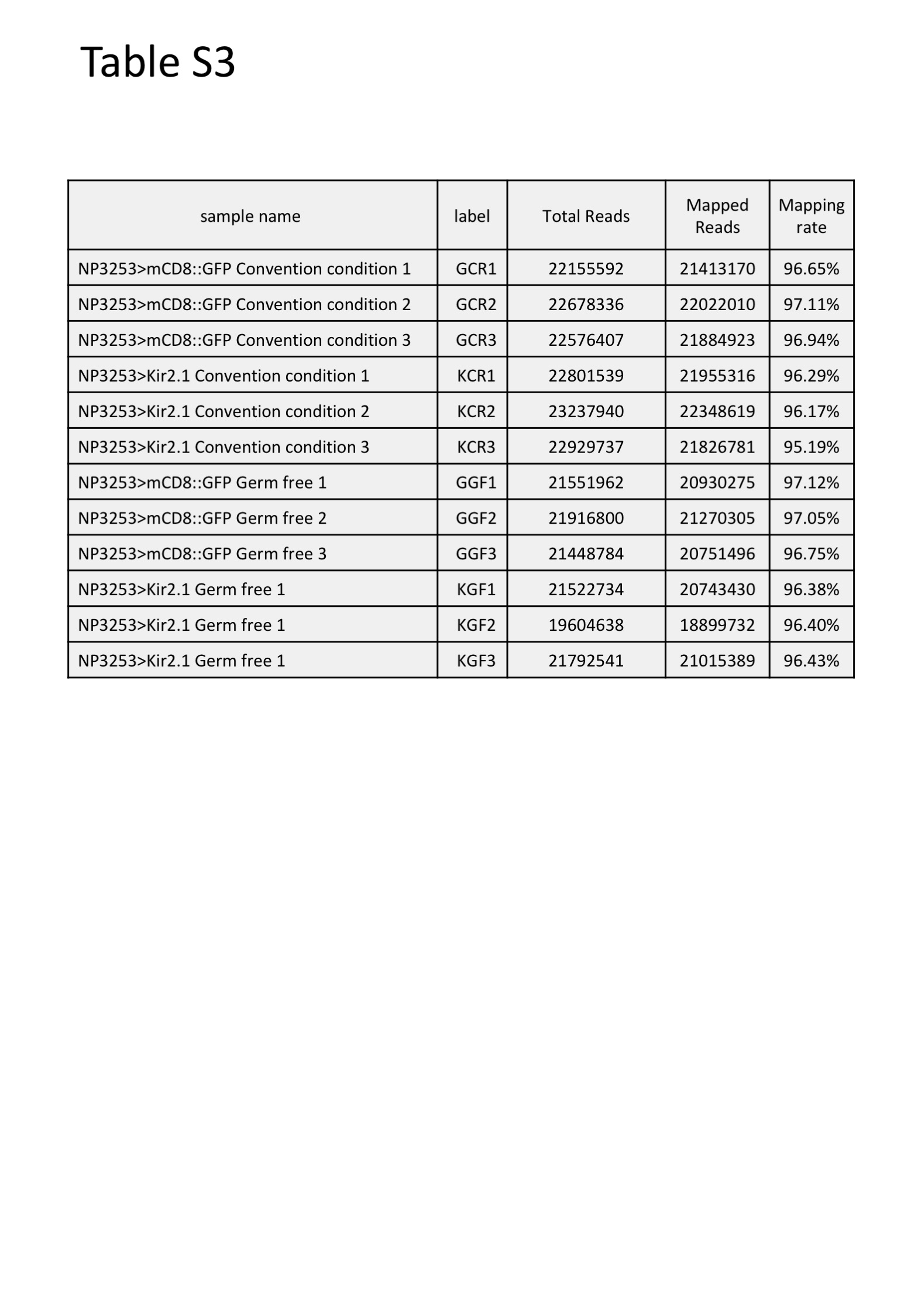

Supplement: Supplementary Table 3 — Summary of the RNA-seq analysis (EXP2). Sample name (genotype, rearing condition, replica number), label (common for Data S4 and DRA012434), total reads, mapped reads, and mapping rates for each sample of RNAseq analysis (EXP2) are indicated. [file Image_8.jpeg]
